# Multiplex immunohistochemistry reveals cochlear macrophage heterogeneity and local auditory nerve inflammation in cisplatin-induced hearing loss

**DOI:** 10.3389/fneur.2022.1015014

**Published:** 2022-10-20

**Authors:** Mai Mohamed Bedeir, Yuzuru Ninoyu, Takashi Nakamura, Takahiro Tsujikawa, Shigeru Hirano

**Affiliations:** Department of Otolaryngology-Head and Neck Surgery, Kyoto Prefectural University of Medicine, Kyoto, Japan

**Keywords:** inner ear, macrophages, multiplex immunohistochemistry, cisplatin, hearing loss

## Abstract

Inner ear macrophages play a vital role in cochlear homeostasis. Recent studies have demonstrated the existence of macrophages at different sites of the cochlea, with increased cochlear infiltration as an inflammatory response mechanism to injury. However, current methods, such as conventional immunohistochemistry and flow cytometry, provide limited information about the diversity of cochlear macrophages. Recently, multiplex immunohistochemistry (mIHC) successfully identified the heterogeneity of immune cells in cancer tissue and thereby improved our understanding of the disease prognosis. In this study, we modified the mIHC technique for cochlear tissue and utilized it to investigate cochlear macrophage behavior and heterogeneity before and after exposure to ototoxic drugs such as cisplatin. Four-week-old C57BL/6N female mice were intraperitoneally injected with cisplatin at 5 mg/kg/day consecutively for 6 days. Their hearing levels were assessed before and after the injection. Their cochleae were harvested before (day 0) and on days 8 and 15 after the cisplatin injection. Paraffin-embedded sections were sequentially immunostained using macrophage surface markers to identify the different categories of macrophages. Each immunostaining cycle included incubation with primary antibody, incubation with secondary antibody, chromogenic staining, and image scanning. Thereafter, all antibodies were stripped out, and antigen retrieval was performed to prepare the tissue for the next cycle. The results revealed that activated cochlear macrophages were not entirely differentiated into M1 or M2 categories but into multi-marker M1/M2 mixed macrophages. Furthermore, the ratio of these mixed (M1/M2) macrophages to Iba1^+^ macrophages increased in the auditory nerve after cisplatin exposure, suggesting local auditory nerve inflammation. The increase in the population of activated macrophages in the auditory nerve region was concomitant with the temporary shift of hearing threshold on day 8 post-cisplatin injection. The findings of this study indicate the effectiveness of mIHC in identifying cochlear macrophage heterogeneity both in the resting state and after cisplatin exposure. Therefore, mIHC could be a powerful tool in cochlear immunology research. Our findings may provide new insights into the co-relation between the cochlear macrophage and cisplatin exposure.

## Introduction

Macrophages play a pivotal role in tissue homeostasis and inflammation, maintaining tissue-specific functions, and protecting organisms from infection. However, they also contribute to the pathophysiology of multiple diseases, including malignant tumors and autoimmune-related diseases (1). Macrophages represent a heterogeneous cell population and display remarkable plasticity in response to distinct microenvironmental stimuli (2). Earlier classifications categorized macrophages into pro-inflammatory macrophages (M1) promoting inflammation, anti-inflammatory macrophages (M2) enhancing tissue repair, and naive or non-activated macrophages (M0) (3, 4). However, these simplistic categories are insufficient for an accurate characterization of macrophage heterogeneity (2, 5, 6). An in-depth analysis of macrophage polarization in the tissue context is essential to better understand the precise mechanisms and physiological functions of macrophages in order to develop future intervention strategies against immune-related diseases.

The inner ear is thought to be immune-free because of the presence of the blood-labyrinth barrier, which restricts the entry of blood cells into the inner ear (7). Nevertheless, recent studies have shown the existence of cochlear immune cells such as resident macrophages in the spiral ganglion, spiral ligament, and stria vascularis (8–12). Cochlear-resident macrophages originate from the yolk sac and fetal liver and are partially replaced by bone marrow-derived circulating monocytes throughout life (12, 13). These macrophages react to cochlear insults, such as noise exposure (10, 14), ototoxic drugs (15), and surgical injuries (16), resulting in their increased local infiltration. Moreover, the cochlear immune cells mediate local innate immune responses by activating the NLRP3 (NOD-, LRR- and pyrin domain-containing protein 3) inflammasome, which is related to regional autoinflammation and progressive hearing loss (17). These findings suggest that macrophages play crucial roles in cochlear homeostasis and hearing function. However, little is known about the precise characterization and physiological functions of the macrophages in the cochlea.

Previous studies investigating the inner ear macrophages have mainly used conventional immunohistochemistry (9, 18, 19), flow cytometry (20), and reverse transcription-quantitative polymerase chain reaction (RT-qPCR) (21). However, these techniques have limitations; hence, they are inadequate for a deeper elucidation of the cochlear macrophage phenotype within the complex tissue. For example, conventional immunohistochemistry is primarily based on single-channel imaging, and immunofluorescence is typically limited to 4–6 channels per tissue slide. Flow cytometry or RT-qPCR provide population or bulk information; hence, they lack the spatial information necessary for analyzing local environmental effects. Recently, multiplex immunohistochemistry (mIHC) and image cytometry techniques have been developed for cancer research and have enabled the quantitative assessment of immune cell heterogeneity with robust spatial information (22, 23). The mIHC technique does not require additional instruments or materials other than those used for the conventional immunohistochemistry but provides more specific information than flow cytometry does (22, 24).

The aim of this study was to optimize the mIHC workflow for vulnerable cochlear tissue. We developed a surface marker panel to investigate the heterogeneity of the cochlear macrophages in the normal state and after exposure to the ototoxic drug cisplatin (CDDP). We successfully performed seven rounds of staining using six different antibodies and hematoxylin without any special reagents or instrumentation. The modified mIHC technique identified multi-marker positive macrophages that were not well polarized to the M1 or M2 axis, suggesting a wide variety of macrophage characteristics in the resting state. Furthermore, the multiplex imaging-based robust spatial information revealed that cisplatin induced macrophage activation and local neuroinflammation associated with temporary hearing threshold shift. These results provided new immunological insights into cisplatin-induced hearing loss. Thus, the modified mIHC method described here is a powerful tool for cochlear immunology and can be effectively integrated into laboratory settings.

## Materials and methods

### Animals

This study was approved (M2021-324) by and conducted according to the established guidelines of the Kyoto Prefectural University of Medicine Animal Center, Japan. Four-week-old C57BL/6N female mice were housed in a specific pathogen-free animal care facility with an independently ventilated cage system, and food and water were provided *ad libitum*. Their circadian rhythm was maintained at 12 h each of light and dark cycles at 23 ± 2°C and 50% ± 10% humidity. The cisplatin and sham-control groups were kept in two separate cages, and numbered ear tags were used to recognize all mice. Age-matched C57L/6N mice were used for each group. The control group mice were inspected first, followed by the cisplatin group. The number of examined cochleae was the same in all groups, unless otherwise indicated in the figure legends.

### Cisplatin injection protocol

Cisplatin was liquefied in saline and injected intraperitoneally at 5 mg/kg/day consecutively for six days until 4 weeks of age, as described previously (25). The control group was injected with saline solution.

### Auditory brainstem response (ABR) measurements

To evaluate the hearing levels of the mice, their ABR was measured on day 0 before the cisplatin injection and on days 7 and 14 after the cisplatin injection. Thus, the ABR of the mice was measured at the age of 4 weeks before the cisplatin injection and at 5 and 6 weeks following it. The mice were prepared for ABR measurements, as described previously (26, 27). Briefly, the mice were anesthetized by intraperitoneal injection of an anesthetic mixture containing medetomidine 0.3 mg/kg, midazolam 4 mg/kg, and butorphanol 5 mg/kg. BioSigRP software (Tucker-Davis Technologies, Alachua, FL, US) and TDT System 3 (Tucker-Davis Technologies, US) were used to determine the ABR measurements. The acoustic stimuli consisted of 8, 16, 24, and 32 kHz tone bursts, presented in a 5 dB step-down sequence from 90 to 20 dB of sound pressure level (dB SPL). ABR waveforms were recorded for 12.8 ms at a sampling rate of 40,000 Hz using a 50–5,000 Hz passband, and 500 responses were averaged at each sound pressure level. The hearing threshold was defined as the lowest sound intensity level at which a detectable wave III was observed. Wave-1 latency was measured as the time between the sound stimuli and a wave peak of a first positive wave at 80 dB SPL.

### mIHC

**Figure 1:**
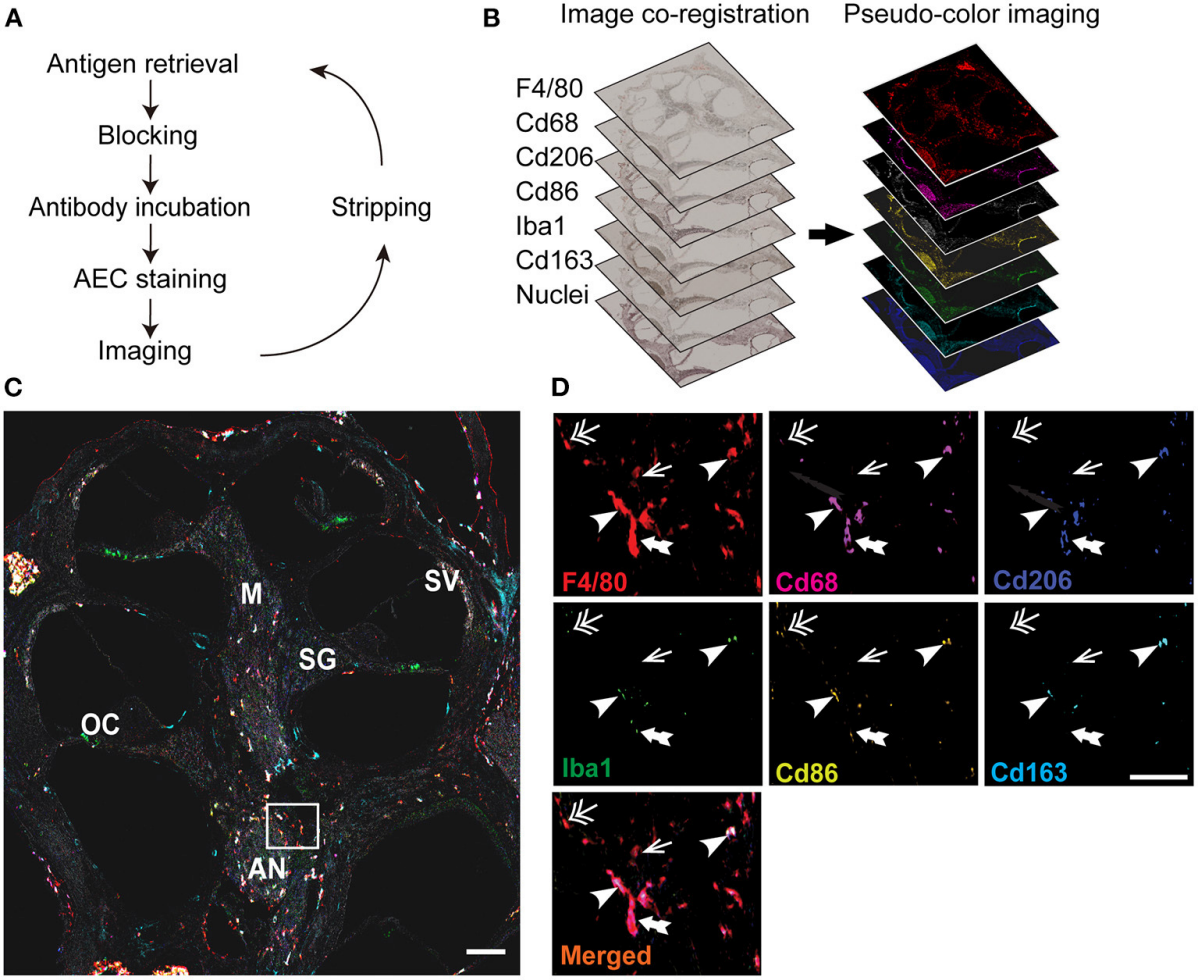
Overview of image processing for multiplex immunohistochemistry (mIHC) of the cochlea. **(A)** Schematic view of the mIHC. The cochlea of 4–6 weeks old mice were harvested, fixed with 4% paraformaldehyde, decalcified, and embedded in paraffin. Sections were deparaffinized and immunostained with a primary antibody and visualized by using chromogenic staining (AEC; 3-amino-9-ethyl-carbazole). After imaging with a whole slide scanner, the antibody and chromogenic stains were stripped out with a serial gradient of ethyl alcohol until the signals disappeared entirely. The staining and stripping steps were repeated for six different macrophage markers (F4/80, CD68, CD206, CD86, Iba1, and CD163) and hematoxylin. **(B)** Image processing. The images were co-registered to align the same area in each slide, and the images were converted into pseudo-colored single-marker images to identify the positive signals of each marker. **(C)** Merged image of all the markers. Individual images were merged into one image showing the expression of all the markers, and analyzed to identify the markers expressed in each F4/80^+^ macrophage. Different areas of the cochlea were examined to identify the macrophage diversity. SG: spiral ganglia, SV: stria vascularis, M: modiolus, OC: organ of Corti, AN: auditory nerve. **(D)** Magnified area showing the markers expressed by the macrophages in the inner ear. Some macrophages expressed only one marker F4/80 (arrow), whereas others expressed four (double-head arrow), five (double-tail arrow), and even all the markers (arrowhead). Scale bar: the whole cochlea, 500 μm; magnified region, 30 μm.

mIHC was performed using sequential cycles of immunostaining. Each cycle consisted of antigen retrieval, blocking, primary and secondary antibodies incubation, chromogenic staining, scanning, and antibody/chromogenic stripping (Figure 1A). The mIHC protocol, initially described in previous studies (22, 23, 28–30), was modified for convenience in cochlear samples as follows. Mice were anesthetized and euthanized by perfusion fixation with 4% paraformaldehyde (PFA) on days 0, 8, and 15, pre- or post-cisplatin injection (**Figure 3A**). Their cochleae were harvested and fixed with 4% PFA in 0.1 M phosphate-buffered saline (PBS; pH 7.4) at 4°C overnight, then decalcified in 0.12 M ethylenediaminetetraacetic acid (EDTA) at 4°C for 3–5 days. Samples were embedded in paraffin blocks, sectioned at 7 μm thickness slices using a Leica RM2125 RT (Leica Biosystems, Nussloch, Germany) microtome, and mounted on adhesive glass slides (CREST, Matsunami Glass Ind., Ltd., Osaka, Japan). The sections were deparaffinized by heating at 63°C for 15 min, immersed in xylene, and hydrated using a series of gradient ethanol (100, 100, 70, and 50%) for 5 min each. After blocking the endogenous peroxidase using 0.6% hydrogen peroxide in 0.1 M PBS at 23°C for 15 min, antigen retrieval was performed using citrate buffer (pH 6.0; LSI Medience Corporation, Tokyo, Japan) in a hot-water bath at 70°C for 5 min, followed by incubation for 10 min at room temperature (Supplementary Figures 1A–C, 2). The sections were then blocked using a blocking buffer [Blocking One Histo solution (Nacalai Tesque, Cat #06349, Kyoto, Japan)] to prevent non-specific binding of antibodies and reduce the background noise during signal detection. The sections were incubated with the primary antibody at 23°C for 1 h and then washed in 0.1 M PBS and double-distilled water. Thereafter, sections were incubated with the secondary antibody at 23°C for 30 min and subjected to chromogenic staining using 3-amino-9-ethyl-carbazole (AEC; SK-4200, Vector Laboratories, CA, US). The whole tissue was scanned using the NanoZoomer S60 Digital slide scanner C13210-01 (Hamamatsu Photonics, Hamamatsu, Japan) at 20× magnification with a pixel resolution of 0.441 μm. The antibody and chromogenic stain were stripped out by serially incubating the tissue section in an increasing gradient of ethanol (50, 70, and 100%) for 1 min each with agitation until the signals disappeared entirely; this step is critical for eliminating the false-positive signals in the subsequent cycles. Additionally, we used primary antibodies raised in different host species in the subsequent cycles to prevent possible false-positive signals (Supplementary Figure 3). A negative control slide was prepared by incubating in 0.1 M PBS, without the primary antibody, for comparison with the positive slides and confirming the true positive signal for each slide. In the final cycle, the tissue sections were stained with hematoxylin (S3301, Agilent, CA, US) at 23°C for 1–3 min. For optimizing above protocol, conventional heat-mediated antigen retrieval methods at 95°C for 15 min with EDTA (pH 8.0) (22) and neutral buffer (pH 7.0) (HistoVT One, Nacalai Tesque, Cat #06380-05, Kyoto, Japan) were tested (Supplementary Figure 1).

### Immunofluorescence staining

The paraffin-embedded cochlear sections were deparaffinized, followed by heat-mediated antigen retrieval with citrate buffer (pH 6.0) at 90°C for 10 min. Sections were blocked with 5% bovine serum albumin (BSA) in 0.3% PBST (PBS, pH 7.2 with 0.3% tween-20) for 1 h followed by incubation with primary antibodies with 3% BSA in 0.03% PBST at 37°C for 2 h. Sections were rinsed with 0.03% PBST and incubated for 30 min at 23°C in species-appropriate secondary antibodies and 4'6-diamidino-2-phenylindole (DAPI) (Dojindo, Cat # D523, Kumamoto, Japan, 1:5000). Stained sections were mounted in ProLong Antifade (Thermo Fischer Scientific Carlsbad, CA, USA) with a coverslip and imaged at x10 and x40 objectives using a confocal microscope (LSM900, Carl Zeiss, Germany).

### Antibodies

Primary antibodies against macrophages were used to identify the different macrophage categories. In brief, all primary antibodies in 0.1 PBS with 0.03% triton-X and 5% blocking solution (Nacalai Tesque) were incubated for 1 h at room temperature (23°C) in the following sequence and concentration: F4/80 monocyte marker for the identification of cochlear macrophages (rat monoclonal, Abcam, Cat # ab6640, RRID # AB_1140040, 1:100) (11); CD68 lysosome-associated membrane protein marker (31) for the detection of inner ear-resident M1 macrophages (rabbit monoclonal, Cell Signaling Technology, Cat # 97778, 1:200) (19); CD206 mannose receptor C type 1 marker for the detection of M2 macrophages (goat polyclonal, R&D systems, Cat # AF2535, RRID # AB_2063012, 1:100) (32); CD86 marker for the detection of M1 macrophages (rabbit monoclonal, Cell Signaling Technology, Cat # 19589, RRID # AB_2892094, 1:100) (33); ionized calcium-binding adaptor molecule 1 (Iba1) microglia/macrophage marker (goat polyclonal, Abcam, Cat # ab5076, RRID # AB_2224402, 1:500) (9), CD163 marker for the detection of M2 macrophages (rabbit monoclonal EPR19518, Abcam, Cat # ab182422, RRID # AB_2753196, 1:200) (34), CXCR1 chemokine receptor (rabbit polyclonal, Bioss antibodies, Cat # bs-1009R, RRID # AB_10857682, 1:250) (35). CD11C dendritic cells marker (rabbit polyclonal, Proteintech, Cat # 17342-1-AP, RRID # AB_2129787, 1:400) (36). TMEM119, microglial marker (rabbit monoclonal, Abcam, Cat # ab209064, RRID # AB_2800343, 1:400) (37) and β3-tubulin marker for investigating neuronal damage (rabbit monoclonal EP1569Y, Abcam, Cat # ab52623, RRID # AB_869991, 1:300) (38). The secondary antibodies included anti-goat antibody and Histofine Simple Stain Mouse MAX-PO (G) (Nacalai Tesque, Cat # 414351), anti-rabbit antibody and Histofine Simple Stain Mouse MAX-PO (R) (Nacalai Tesque, Cat # 414341), and anti-rat antibody and Histofine Simple Stain Mouse MAX-PO (rat) (Nacalai Tesque, Cat # 414311). For immunofluorescence staining, Alexa Fluor 488-conjugated anti-rabbit IgG (RRID # AB_143165; 1:500) and Alexa Fluor 564-conjugated anti-goat IgG (RRID # AB_2534103; 1:500) were obtained from Thermo Fischer Scientific (Waltham, MA, USA). All secondary antibodies were incubated for 30 min at room temperature.

### Macrophage quantification

**Figure 2:**
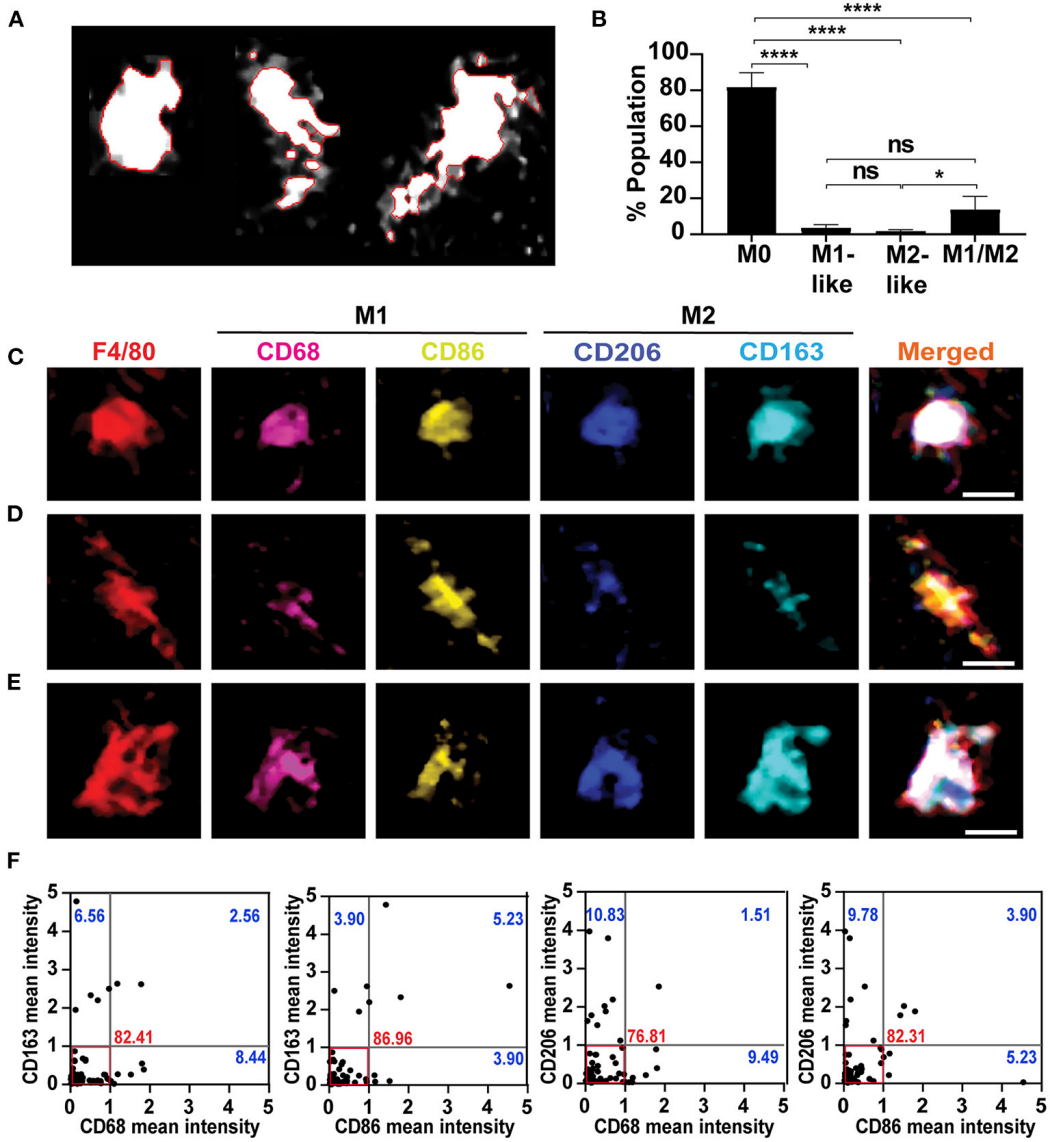
Classification of mixed macrophages according to the intensity of expressed markers**. (A)** Watershed cell segmentation to identify the boundaries of single macrophages. Cochlea were obtained from 4-weeks old mice, and immunostained using the mIHC. The red line denotes the boundaries of each macrophage with F4/80 staining. **(B)** Quantification of the population in the resting state to identify the percentage of each category in the modiolus including auditory nerve and spiral ganglia. Mid-modiolar sections were analyzed, and macrophages population of each category, M0, M1-like, M2-like and M1/M2, were quantified (*n* = 5). The M0 non-activated macrophages represented the major percentage among all macrophages, with a significant difference compared to other categories. The mixed M1/M2 macrophages were presented minimally, while the M1-like and M2-like macrophages expressing single marker represented the least percentages. *P < 0.05, ****P < 0.0001, ns: not significant, by one-way ANOVA with Tukey's multiple comparisons test. The error bars represent standard deviation from the mean. **(C)** Example of mixed macrophages in which M1 markers (CD68, CD86) expression was similar to that of the M2 markers (CD206, CD163). **(D)** A representative macrophage of a group of mixed macrophages in which the CD86 (M1 marker) showed the highest expression compared to the other markers. **(E)** Illustration of another group of mixed macrophages in which CD163 (M2 marker) had the highest expression among all markers. **(F)** Single-cell-based marker intensity for each mixed macrophage in the modiolus including auditory nerve and spiral ganglia. Each dot represents a single mixed macrophage (*n* = 74 from 7 cochleae); the mean intensity of M1 and M2 markers in each macrophage are plotted against each other. The cut-off value was set at 1, below which most of the macrophages were included, and the different types of macrophages were identified. The red box marks the area of the dominant cell population in which most of the macrophages represent M1 and M2 markers equally within the cut-off value. The numbers indicate the percentages of the cell population of each quadrant; in the lower right quadrant, M1-polarized cells expressing higher M1 marker intensities (>1) than M2 cells, 8.44, 3.90, 9.49, and 5.23%: the upper left quadrant, M2-polarized cells expressing higher M2 marker intensities (>1) than M1 cells, 6.56, 3.90, 10.83, and 9.78%: the upper right quadrant, cells strongly expressing both M1 and M2 markers, 2.56, 5.23, 1.51, and 3.90%.

Image processing included three steps: image co-registration, image visualization, and image analysis. For image co-registration, the images were co-registered at a single-pixel level using the CellProfiler v.2.1.1 pipeline Alignment_Batch.cppipe (available under General Public License version 2 at https://github.com/multiplexIHC/12plex-IHC/blob/master/Alignment%20-%20Batch.cppipe) so that the same area was aligned for all images (Figure 1B). The image visualization consisted of converting the co-registered images into pseudo-colored single-marker images using ImageJ v.1.48 (Figure 1B) (available at https://imagej.nih.gov/ij/) and Aperio ImageScope v12.4.3.5008 (Leica Biosystems, Germany; available at https://aperio-imagescope-x64.software.informer.com/12.4/), as described previously (22). All the images were then merged into one final image showing the expression of all macrophage markers (Figure 1C). The final step was image analysis, wherein the macrophages in the central region of the cochlea, including the modiolus, spiral ganglia, and auditory nerve, and additional macrophages in the stria vascularis and organ of Corti were quantified using ImageJ v.1.48. The markers expressed by each macrophage were identified, and the macrophages were categorized accordingly (Table 1). Watershed cell segmentation was performed using ImageJ v.1.48 (available at https://imagej.net/plugins/morphological-segmentation) to identify each macrophage border (Figure 2A). This step was followed by colocalization analysis of each macrophage, to compare the intensity of markers against one another, using ImageJ v.1.48 (available at https://imagej.net/plugins/coloc-2) (Figure 2F). The proportions of CD68^+^, CD86^+^, CD206^+^, CD163^+^, and Iba1^+^ macrophages represent the ratio of macrophages positive for each marker to F4/80^+^ macrophages or total number of macrophages (**Figure 4B**).

**Table 1:** Categorization of macrophages according to their expressed markers.

**Category**	**Identification marker**
M0 (non-activated)	F4/80^+^
M1 (pro-inflammatory)	F4/80^+^ + CD68^+^
	F4/80^+^ + CD86^+^
	F4/80^+^ + CD68^+^ + CD86^+^
	F4/80^+^ + CD68^+^ + CD86^+^+ Iba1^+^
M2 (anti-inflammatory)	F4/80^+^ + CD206^+^
	F4/80^+^ + CD163^+^
	F4/80^+^ + CD206^+^ + CD163^+^
	F4/80^+^ + CD206^+^ + CD163^+^ + Iba1^+^
M1/M2 (mixed)	F4/80^+^ + CD68^+^ + CD86^+^ + CD206^+^ + CD163^+^ + Iba1^+^
	F4/80^+^ + CD86^+^ + CD206^+^ + CD163^+^ + Iba1^+^
	F4/80^+^ + CD68^+^ + CD206^+^ + CD163^+^ + Iba1^+^
	F4/80^+^ + CD68^+^ + CD86^+^ + CD163^+^ + Iba1^+^
	F4/80^+^ + CD68^+^ + CD86^+^ + CD206^+^ + Iba1^+^
	F4/80^+^ + CD68^+^ + CD86^+^ + CD206^+^ + CD163^+^

M0: macrophages expressing only F4/80. M1: macrophages expressing one or two M1 markers only. M2: macrophages expressing one or two M2 markers only. Mixed: macrophages expressing all markers or at least one M1 marker and one M2 marker.

### Neuronal cell count

To investigate the state of the spiral ganglia neurons and auditory nerve, we stained the same paraffin sections with a β3-tubulin neuronal marker at room temperature for 1 h. Neuronal cells were counted using ImageJ v.1.48., images of the cells expressing β3-tubulin only were converted into 8-bit monochrome type, and the desired area was then analyzed.

### Hair cell count

For calculating the outer hair cell (OHC) and inner hair cell (IHC) survival after cisplatin injection, the cochleae were fixed with 4% PFA in 0.1 M PBS at room temperature for 2 days, followed by decalcification in 0.12 M ethylenediaminetetraacetic acid at room temperature for 3–5 days. The cochleae were then sectioned into three turns: apical, middle, and basal. Immunostaining was performed using phalloidin (1:400) at room temperature for 1 h. Images were acquired using a confocal microscope (LSM900) at 10× magnification with a 1.4× digital zoom. Each image contains at least 20 adjacent inner hair cells. The hair cells were counted manually, and the damaged hair cells were identified by the absence of phalloidin staining and the presence of gaps between the adjacent cells. The average ratio of the remaining cells was calculated for each group.

### Statistical analysis

All data are presented as mean ± standard deviation. Significant differences between the animal groups were detected using a one-way or two-way analysis of variance (ANOVA) depending on the evaluated parameter, followed by an appropriate *post hoc* test. The results were considered statistically significant at *P* < 0.05 (^*^), *P* < 0.01 (^**^), *P* < 0.001 (^***^), and *P* < 0.0001 (^****^). All statistical analyses were performed using the Prism 9.2.0 software (GraphPad Software Inc., La Jolla, CA, USA). All statistical details, including the exact n and *p* value, and which statistical test was performed, can be found in the figure legends.

## Results

### Identification of macrophage heterogeneity in the cochlear sample using mIHC and digital imaging of formalin-fixed paraffin-embedded (FFPE) sections

To investigate the heterogeneity of cochlear-resident macrophages, we optimized the mIHC protocol (Figure 1A) and developed a macrophage surface marker panel consisting of six different antibodies: F4/80 for the monocyte-origin macrophages, CD68 and CD86 for the M1 or pro-inflammatory macrophages, CD206 and CD163 for the M2 or anti-inflammatory macrophages, and Iba1 for the microglial macrophages (Figure 1B). The epitopes were well conserved throughout the sequential cycles; hence, mIHC helped visualize multiple surface markers with precise anatomical architecture in single cochlear FFPE sections (Figure 1C). Notably, macrophages labeled with these markers were dominant in the central cochlear region. The cochlear macrophages expressed a variety of markers; thus, macrophages were single marker-positive or F4/80^+^, tetra-marker-positive or F4/80^+^ CD68^+^ CD86^+^ Iba1^+^, penta-marker-positive or F4/80^+^ CD68^+^ CD86^+^ CD206^+^ Iba1^+^, and hexa-marker-positive or F4/80^+^ CD68^+^ CD86^+^ CD206^+^ CD163^+^ Iba1^+^ (Figure 1D). Based on these phenotypes, we categorized the macrophages into M0, M1, M2, and M1/M2 mixed macrophages (Table 1) (34). These results reveal the ability of the optimized mIHC protocol to identify the different variants of inner ear macrophages distinctly.

### Heterogeneity of M1/M2 mixed macrophages identified by mIHC and quantification of single-cell-based chromogenic intensities of multiple macrophage markers

To explore the M1/M2 mixed macrophage variants, we measured the chromogenic intensities of each M1 (CD68 or CD86) or M2 (CD163 or CD206) marker in the serial AEC-stained images, and analyzed their co-expression levels in each mixed macrophage at the modiolus including auditory nerve and spiral ganglia using the cell segmentation (Figure 2A) and the colocalization plugin of Image J software. This single-cell-based multi-parametric information revealed a diversity of cochlear mixed macrophages expressing heterogeneous M1 and M2 markers. The M0 non-activated macrophages majorly represented the cell population (81.57 ± 8.12%), followed by the M1/M2 mixed (13.50 ± 7.50%), M1 (3.36 ± 2.04%), and M2 (1.55 ± 0.96%) macrophages, with significant difference between the ratio of M0 and the other categories (*P* < 0.0001 with M0 vs. M1-like, M0 vs. M2-like, and M0 vs. M1/M2; *P* < 0.05 with M2 vs. M1/M2) (Figure 2B). The mixed macrophages were subdivided into three groups, expressing M1 and M2 markers equally (Figure 2C), M1 marker dominantly (Figure 2D), and M2 marker dominantly (Figure 2E). Most mixed macrophages expressed the M1 and M2 markers equally and represented the majority of the macrophage population (Figure 2F; 82.41 ± 7.43% with CD68 vs. CD163, 86.96 ± 11.51% with CD86 vs. CD163, 76.81 ± 7.02% with CD68 vs. CD206, and 82.31 ± 10.43% with CD86 vs. CD206), while few mixed macrophage populations showed a higher intensity of the M1 marker (8.44 ± 4.06% with CD68 vs. CD163, 3.90 ± 3.26% with CD86 vs. CD163, 9.49 ± 1.89% with CD68 vs. CD206, and 5.23 ± 5.01% with CD86 vs. CD206) or of the M2 marker (6.56 ± 6.83% with CD163 vs. CD68, 3.90 ± 3.26% with CD163 vs. CD86, 10.83 ± 3.71% with CD206 vs. CD68, and 9.78 ± 4.34% with CD206 vs. CD86). Other mixed macrophages strongly expressed both M1 and M2 markers more than the cut-off value (Figure 2F; 2.56 ± 1.81% with CD68 vs. CD163, 5.23 ± 5.01% with CD86 vs. CD163, 1.51 ± 2.14% with CD68 vs. CD206, and 3.90 ± 3.26% with CD86 vs. CD206). These results show a diversity of resident macrophage characteristics, even in the resting state.

### Cisplatin activated macrophages in the auditory nerve region

**Figure 3:**
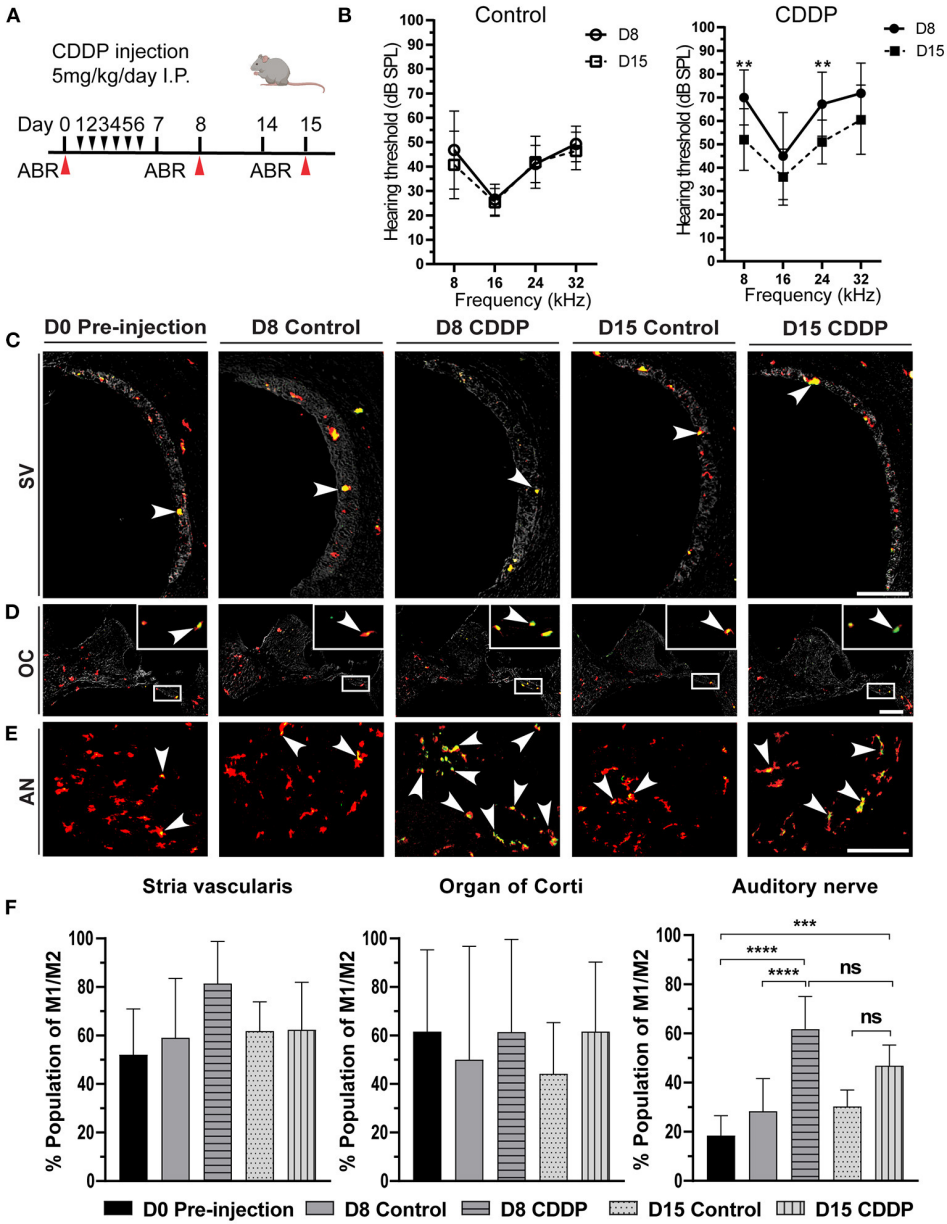
Cisplatin induced temporary hearing threshold shift and macrophage activation. **(A)** The protocol for cisplatin injection (red arrows indicates the time of euthanasia). **(B)** Auditory brain stem response (ABR) thresholds [dB sound pressure level] at 8, 16, 24, and 32 kHz in the control and cisplatin groups of 5-week-old mice just after cisplatin injection (day 8) and after 2 weeks (day 15). There was a temporary threshold shift at day 8 (*n* = 14), which significantly improved at day 15 (*n* = 10) at 8 kHz (*P* = 0.0056), and 24 kHz (*P* = 0.0166) but was insignificant at 16 kHz (*P* = 0.4854) or 32 kHz (*P* = 0.1908). There was no significant difference between the hearing thresholds of the control groups (*n* = 14 for each group). Statistical analysis was done by two-way ANOVA with the Bonferroni *post hoc* test. The error bars represent standard deviation from the mean. **(C)** Activated macrophages in the stria vascularis. **(D)** Activated macrophages in the organ of Corti. The box shows the magnified region of the macrophages in the organ of Corti. **(E)** Activated macrophages in the auditory nerve. The arrowhead represents the activated macrophages (yellowish green color). There was a marked increase in the activated macrophages of the auditory nerve area on day 8 post-cisplatin injection. However, this activation was temporary, and the number of activated macrophages was reduced on day 15. AN: auditory nerve, SV: stria vascularis, OC: organ of Corti. Scale bar: stria vascularis, 90 μm; Organ of Corti, 200 μm; auditory nerve, 40 μm. **(F)** Statistical analysis of the ratio of activated macrophages in different sites of the cochlea. In the Stria vascularis and organ of Corti, there was no significant difference in the amount of activated macrophages between the different groups. In the auditory nerve, there was significant increase in the number of activated macrophages on day 8 (*n* = 10). This activation was insignificantly reduced on day 15 (*n* = 8), ***P < 0.001, ****P < 0.0001, ns: not significant, by one-way ANOVA with Tukey's multiple comparisons test. The error bars represent standard deviation from the mean.

The ABR of the mice was measured before and after the cisplatin injection to examine its effects on hearing threshold. The cisplatin-injected mice showed a temporary shift of hearing threshold (control vs. cisplatin on day 8: 46.78 ± 16.00 dB vs. 70.00 ± 11.76 dB, 26.42 ± 6.33 dB vs. 45.00 ± 18.60 dB, 41.07 ± 7.64 dB vs. 67.14 ± 13.68 dB, and 49.28 ± 7.30 dB vs. 71.78 ± 12.95 dB at 8, 16, 24, and 32 kHz respectively; control vs. cisplatin on day 15: 40.71 ± 13.84 dB vs. 52.00 ± 13.16 dB, 25.35 ± 5.70 dB vs. 36.00 ± 11.97 dB, 41.78 ± 10.67 dB vs. 51.00 ± 9.36 dB, and 46.42 ± 7.70 dB vs. 60.50 ± 14.80 dB at 8, 16, 24, and 32 kHz respectively). There were significant differences in the hearing threshold between days 8 and 15 at 8 kHz (*P* = 0.0056), and 24 kHz (*P* = 0.0166) but not at 16 kHz (*P* = 0.4854) and 32 kHz (*P* = 0.1908) in the cisplatin group (Figure 3B). However, there was no significant loss of spiral ganglion and auditory nerve [control vs. cisplatin at day 15: 98.00 ± 51.38 vs. 91.00 ± 22.97 (*P* > 0.9999) in the spiral ganglion and 225.60 ± 64.96 vs. 149.80 ± 47.72 (*P* = 0.0546) in the auditory nerve; Supplementary Figures 5A,B)]. Additionally, no significant OHC and IHC losses were observed in the mice of the cisplatin group [control vs. cisplatin on day 15 at apical, middle, and basal: 99.44 ± 0.96% vs. 100%, 99.16 ± 1.67% vs. 99.58 ± 0.83%, and 99.16 ± 1.18% vs. 97.77 ± 3.85%, respectively, in the OHC (*P* > 0.9999 in all turns) and 100 vs. 100% in the IHC at all turns; Supplementary Figures 5C,D].

In the resting state of the cochlea, most macrophages were in the M0 state, with a minor ratio of activated M1/M2 mixed macrophages (Figure 2B). Consequently, we investigated the effect of cisplatin on macrophage behavior at different sites in the cochlea. On day 8, there was a significant increase of the M1/M2 mixed macrophage population in the auditory nerve region of the mice in the cisplatin group (control vs. cisplatin on day 8: 28.34 ± 13.25% vs. 61.80 ± 13.24%; *P* < 0.0001; Figures 3E,F). This increase of the M1/M2 mixed macrophage population was temporary and showed recovery on day 15 (day 8 cisplatin vs. day 15 cisplatin; *P* = 0.0679), (control vs. cisplatin on day 15: 30.25 ± 6.71% vs. 46.86 ± 8.43%, *P* = 0.0653). Moreover, there was no significant difference in the population of activated macrophages either in the stria vascularis or in the organ of Corti [control vs. cisplatin on day 8: 59.06 ± 24.46% vs. 81.49 ± 17.32% (*P* = 0.2443) in the stria vascularis and 50.00 ± 46.77% vs. 61.42 ± 38.15%, (*P* = 0.9810) in the organ of Corti; Figures 3C,D,F]. The increase in activated macrophages in the auditory nerve region was concomitant with the temporary shift of hearing threshold on day 8. These results suggest local changes in the macrophage microenvironment of the auditory nerve due to cisplatin exposure, resulting in the activation of M1/M2 mixed macrophages.

### Cisplatin-induced overexpression of the microglial marker Iba1 in M1/M2 mixed macrophages

**Figure 4:**
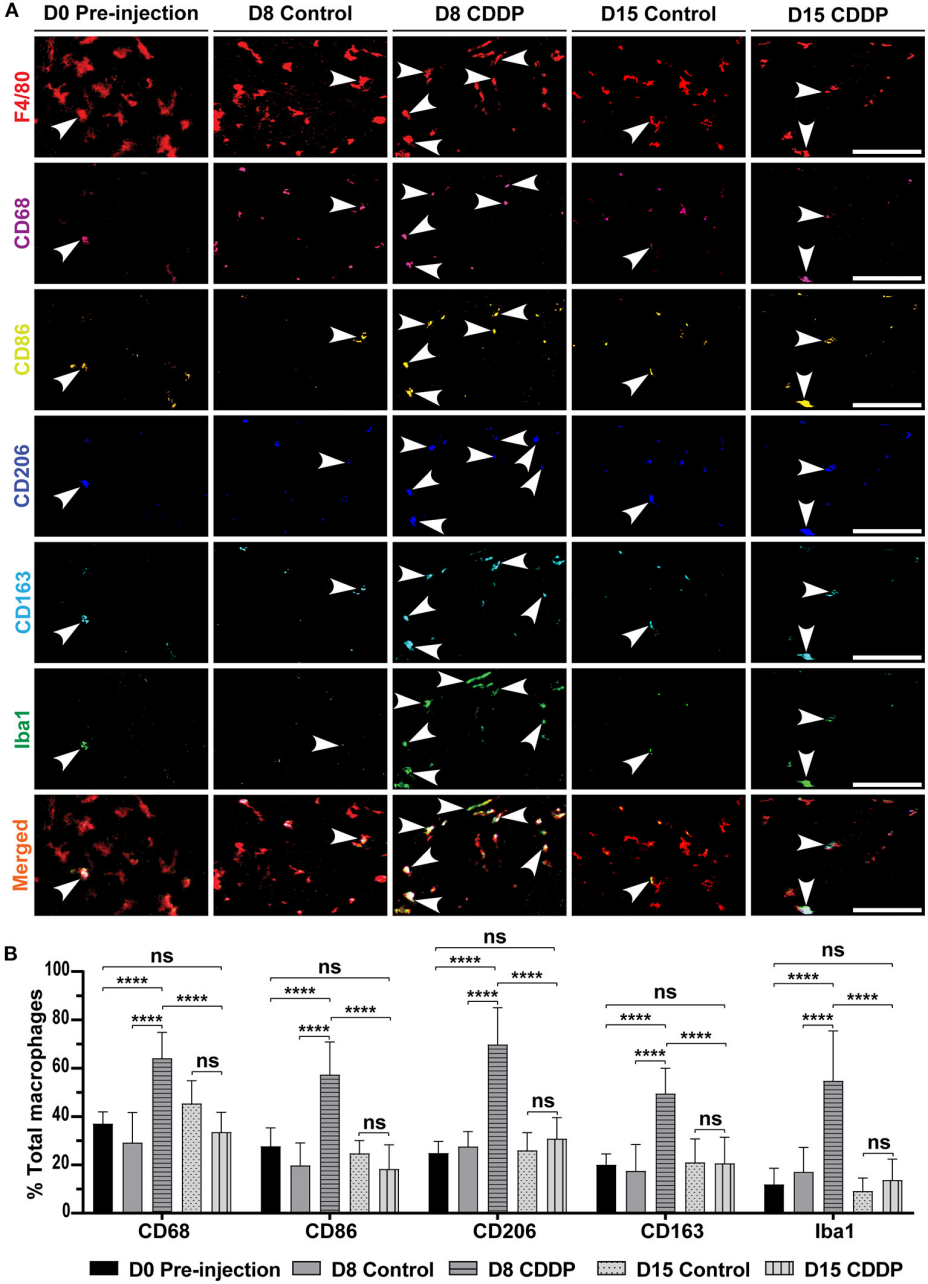
Cisplatin increases the expression of M1, M2, and Iba1 markers in the auditory nerve. **(A)** Macrophage marker expression in different groups. On day 8 post-cisplatin injection, there was a marked increase in the expression of M1 markers (CD68 and CD86), M2 markers (CD206 and CD163), and Iba1. This increased expression was temporary and showed recovery on day 15. This increased expression suggested a temporary auditory nerve inflammation. There was no difference between the expressed markers in the control group. **(B)** Statistical analysis of the ratio of each marker expression in F4/80^+^ macrophages. In all the markers, there was significant increase in the ratio of expression on day 8 in cisplatin-injected mice (*n* = 10) compared to that of the day 8 control (*n* = 8), day 0 pre-injection (*n* = 6), and day 15 cisplatin-injected mice (*n* = 8). ****P < 0.0001, ns, not significant by two-way ANOVA with Bonferroni *post hoc* test. The error bars represent standard deviation from the mean.

To understand how the cisplatin-induced activated macrophages act in the auditory nerve region, we investigated the markers expressed in the M1/M2 mixed macrophages on days 8 and 15. On day 8, the expression of all M1 and M2 markers increased in the cisplatin group (Figure 4A) [control vs. cisplatin on day 8: 29.155 ± 12.48% vs. 64.05 ± 10.77% (*P* < 0.0001) in CD68, 19.63 ± 9.44% vs. 57.25 ± 13.59% (*P* < 0.0001) in CD86, 27.52 ± 6.21% vs. 69.69 ± 15.31% (*P* < 0.0001) in CD206, and 17.39 ± 11.05% vs. 49.44 ± 10.54% (*P* < 0.0001) in CD163]. Moreover, on day 8, the expression of the microglial marker Iba1 significantly increased in the mice of the cisplatin group (control vs. cisplatin on day 8: 16.98 ± 10.25% vs. 54.67 ± 20.75%; *P* < 0.0001). Iba1 expression significantly reduced by day 15, resulting in no significant difference between the Iba1 levels of the mice in either groups [control vs. cisplatin on day 15: 45.29 ± 9.51% vs. 33.46 ± 8.25% (*P* = 0.3227) in CD68, 24.66 ± 5.39% vs. 18.20 ± 10.07% (*P* > 0.9999) in CD86, 25.89 ± 7.42% vs. 30.77 ± 8.76% (*P* > 0.9999) in CD206, 20.89 ± 9.84% vs. 20.55 ± 10.86% (*P* > 0.9999) in CD163, and 9.04 ± 5.46% vs. 13.62 ± 8.75% (*P* > 0.9999) in Iba1; Figure 4B]. This increase in marker expression was not associated with significant neuronal loss of the auditory nerve and the spiral ganglia (Supplementary Figures 5A,B). These results illustrate that acute exposure to cisplatin temporarily activated the macrophages and induced the expression of the microglial marker Iba1 by the M1/M2 mixed macrophages of the auditory nerve region, suggesting latent neuronal inflammation.

## Discussion

In this study, we investigated cochlear-resident macrophage heterogeneity using the novel technique of sequential mIHC. The results revealed macrophage polarization and changes in cisplatin-induced hearing loss. The cochlea is considered an immune-free organ owing to its anatomical features (7). However, recent studies have revealed the existence of yolk sac-derived cochlear-resident macrophages and implicated their crucial role in maintaining cochlear homeostasis (13). Moreover, macrophages are firmly imprinted by their tissue residence or microenvironment (39, 40). An in-depth analysis of these characteristics at the site is essential for understanding the physiological and pathological functions of cochlear immune cells.

The mIHC technique was developed in cancer research and forms a link between flow cytometry and conventional immunohistochemistry. It enables a multi-marker investigation of immune cells for obtaining vital spatial information (23, 30). However, its utilization for vulnerable cochlear tissue is challenging because of the damage caused by repeated stripping and antigen retrieval steps, which are essential for eliminating residual antibodies and restoring the epitope-antibody binding viability (Supplementary Figure 2). This study optimized the protocol for mIHC of the cochlear FFPE sections using citrate buffer (pH 6.0) and a mild heating method for antigen retrieval to preserve the cochlear structure from damage due to the sequential stripping and antigen retrieval steps (Supplementary Figures 1A–C). In addition, we adopted a six-marker chromogenic mIHC platform with tissue segmentation algorithms, enabling the identification of resident macrophages with complete spatial information (Figure 1C). The use of six antibodies for the marker panels in this study does not indicate a limitation of the number of iterative cycles for the cochlear tissue (Supplementary Figure 1C). A previous study used a multiplex panel of 12 markers and confirmed the viability of tissues after frequent cycles of immunostaining (22). Recently, other iterative IHC methods (41–43), such as iterative bleaching extends multiplexity (IBEX), have been reported, which enable high-level multiplexing of protein and nucleic acid detection using multiple fluorophores and chemical bleaching (44). However, these fluorophore-based iterative immunohistochemistry methods rely on the quality and number of available conjugated antibodies or on a time-consuming quenching step. Additionally, considering the clinical settings and developing techniques for a wider availability, our chromogenic mIHC technique is simpler and more feasible for analyzing clinical FFPE samples without any sample modifications and expensive apparatus. Therefore, this novel mIHC technique may be a powerful tool in cochlear research.

In contrast to earlier studies that categorized macrophages into either M1 or M2 (4) activated phenotypes, mIHC revealed M1/M2 mixed macrophages in the cochlea. These macrophages are not well polarized into the M1 and M2 axes, and we detected three subcategories of these M1/M2 mixed macrophages according to their signal intensities (Figures 2C–E, Supplementary Figure 4). These “mixed” macrophages were reported in systemic sclerosis-related interstitial lung disease, melanoma, and hyperglycemia (34, 45, 46). To the best of our knowledge, this is the first study to describe cochlear-resident macrophages, indicating a wide variety of macrophages in the inner ear.

Mixed macrophages represent a minor proportion of F4/80^+^ macrophages in the resting state. In contrast, on day 8 after the cisplatin exposure, mixed macrophages comprised the major subpopulation among the F4/80^+^ macrophages (Figures 3E,F). Furthermore, a considerable number of studies have revealed that differentiation of cochlear-resident macrophages occurs after exposure to damage (8, 10, 18, 21, 46). Consequently, these results suggest the presence of stimulant signals that induce the differentiation of macrophages, suggesting a state of cochlear inflammation. Interestingly, there was a remarkable difference in macrophage differentiation before and after cisplatin exposure only in the auditory nerve region, but not in the stria vascularis and organ of Corti (Figures 3C–E). Additionally, on day 8 after cisplatin exposure, there was a significant increase in Iba1^+^ macrophages or microglia-like macrophages in the auditory nerve area (Figures 4A,B), corresponding to the temporary ABR threshold shift without hair cell loss (Supplementary Figures 5C,D), and suggesting acute latent auditory nerve inflammation. Indeed, we detected wave 1 latency of ABR at 24 and 32 kHz significantly delayed compared to the control group (Supplementary Figure 5E). Earlier studies have shown that cochlear insults, such as surgery (9), noise (47), chemotherapy (48, 49) or oxaliplatin (50–54), can induce transient elevated Iba1 expression, implicating local stress and acute inflammation. Moreover, in some susceptible cases, macrophages become ready for any environmental stimulus and become more activated, leading to cochlear damage (21, 55). These results suggest that acute exposure to cisplatin induces local neuronal inflammation and temporary macrophage activation in the auditory nerve region.

To date, the polarization of cochlear macrophages in response to environmental stimuli has been controversial. On the one hand, Dijkgraaf et al. showed that cisplatin induced M2-polarized macrophages (56). On the other hand, Okano showed that when macrophages are exposed to an area of injury, they are stimulated to produce inflammatory mediators and polarize toward the M1 axis (12). In our multiplex analysis, the mixed macrophages on day 8 tended to be more M2-polarized (Figure 4). However, these macrophages were not clearly divided into M1 and M2 categories and showed a wide variety of surface marker expression (Figure 4A). These data indicate that the simple conventional two-axis model is insufficient to explain the complexity of the physiological role of macrophages. Indeed, macrophages show high plasticity to polarize according to the signal in their microenvironment (57). To elucidate the physiological role of the diverse mixed macrophages, further studies are needed using not only the surface but also functional macrophage markers or cytokine panels. In addition, to overcome the non-linear staining intensity of chromogenic immunohistochemistry for semi-quantitative analysis (58), validating the image cytometry workflow for cochlear tissue is essential (22).

In order to exclude the gender effect, we only analyzed female mice of C57BL/6N, which might be a limitation of this study. Recently, sex differences in ototoxicity have been widely discussed, and sexual dimorphism should be considered in translational neuroscience (59, 60). In species such as Wistar albino rats (61) and C57BL/6J mice (62), the female is more sensitive to cisplatin than the male. However, an estrogen-mediated neuroprotection pathway against cisplatin was reported in CBA/CaJ mice (63). Hence the gender effect on ototoxicity is still controversial. Further investigation is needed to elucidate a gender effect on the cochlear macrophage activation induced by cisplatin.

We utilized the same injection protocol of cisplatin treatment as described previously (25); however, our mice showed less permanent threshold shift of ABR and HCs loss than in the previous paper. It could be explained by the difference in age-related susceptibility to cisplatin (4-week vs. 2-month old). Additionally, the sex population and substrains of C57BL/6 were not identical either. Indeed, aging increases the nephrotoxicity of cisplatin in Sprague-Dawley rat (64), and different susceptibility to noise exposure and aminoglycoside antibiotics between C57BL/6 strains was reported (65). For better understanding of the effect of the immunomodulation by cisplatin on hearing loss, a more reproducible and clinically relevant mouse model of ototoxicity should be utilized in future study (66–68).

In summary, we successfully introduced mIHC to cochlear tissue and reported cochlear macrophage heterogeneity both in the resting state and after exposure to cisplatin. In addition, the mIHC could distinctly visualize six different macrophage surface markers with complete spatial information. Interestingly, there were mixed M1/M2 macrophages in the resting state cochlea, and most of the macrophages were not well-polarized and exhibited both M1 and M2 markers in different amounts. Moreover, these mixed macrophages temporarily increased in number after cisplatin injection, suggesting local macrophage activation and auditory nerve inflammation due to cisplatin exposure. Therefore, mIHC could be a powerful tool in cochlear immunology research, and our findings may provide new insights into the correlation between macrophage activation and cisplatin ototoxicity.

## Data availability statement

The original contributions presented in the study are included in the article/Supplementary material, further inquiries can be directed to the corresponding author.

## Ethics statement

The animal study was reviewed and approved by the Animal Facility of Kyoto Prefectural University of Medicine.

## Author contributions

Study conceptualization, had full access to all the data in the study, takes responsibility for the integrity of the data, accuracy of the data analysis, and validation: YN. Supervision: TT and SH. Investigation and funding acquisition: MB, YN, and TN. Formal analysis: MB. Visualization and writing-original draft: MB and YN. All authors reviewed and edited the important contents of the manuscript.

## Funding

This study was supported by grants from the Japanese Society for the Promotion of Science (JSPS) KAKENHI (JP20K18321 to YN and JP21K09638 to TN) and the Society for Promotion of International Oto-Rhino-Laryngology (SPIO) (SR21002 to MB). One of our authors (MB) is supported by Otsuka Toshimi Scholarship Foundation (OTSF).

## Conflict of interest

The authors declare that the research was conducted in the absence of any commercial or financial relationships that could be construed as a potential conflict of interest.

## Publisher's note

All claims expressed in this article are solely those of the authors and do not necessarily represent those of their affiliated organizations, or those of the publisher, the editors and the reviewers. Any product that may be evaluated in this article, or claim that may be made by its manufacturer, is not guaranteed or endorsed by the publisher.

## Supplementary material

Supplementary Figure 1 Optimization of the iterative antigen-retrieval and primary-antibody incubation steps for the mIHC protocol. Comparison of cochlear tissue damages and background staining among conventional heat-mediated antigen retrieval (AR) at 95°C for 15 min with EDTA (pH 8.0) **(A)**, with neutral buffer (pH 7.0) **(B)**, and mild-heating AR with citrate buffer (pH 6.0) at 70°C for 5 min **(C)**. **(A)** Minor-tissue damage was detected on the first cycle (arrow) and the section started to be detached after the third cycle (arrow head). Note that intense tissue damage was observed in the modiolus (asterisk), resulted in overstaining of hematoxylin. **(B)** Tissue-damage was observed from the third cycle (black arrow heads) and most of the cochlea was destroyed after the fifth cycle (arrow and double arrow). Non-specific signaling in the modiolus at the fourth cycle (asterisk) and overstaining of hematoxylin were observed. **(C)** The cochlear tissue was well-preserved and no major damage was detected up to the end of all the desired staining cycles with clear background. Nuclei were clearly stained by hematoxylin at ninth cycle. **(D)** Short primary antibody incubation reduced the undesired background. 1-hour incubation at 23°C (upper row) and overnight incubation at 4°C (lower row) with CD163 antibody were shown, and each boxed region was magnified in the right panel. Arrow heads indicated positive signal of CD163 with clear background. Scale bar; **(A–C)** 500 μm, **(D)** the whole cochlea 500 μm, the magnified part 200 μm.

Supplementary Figure 2 Mild antigen retrieval protocol removes antibodies entirely and effectively. **(A)** Image of the tissue incubated with the F4/80 antibody; the red dots represent a positive signal. **(B)** Negative control slide incubated with 0.1 M phosphate-buffered saline (PBS); no signal is observed. **(C)** The same tissue in image **(A)** after antigen retrieval, followed by incubation with secondary antibody to check for a residual primary antibody signal. The image shows complete stripping of the primary antibody, indicated by the absence of a positive signal. **(D)** The negative control slide shows no difference after antigen retrieval. Scale bar: 500 μm.

Supplementary Figure 3 Antibodies raised in different host species are used in the sequential immunostaining cycles to confirm the absence of false-positive signals. The images show six immunostaining cycles in the same tissue section. Each cycle of primary antibody incubation was followed by a cycle of incubation with another primary antibody of different species, to avoid a false-positive signal from frequent staining. Scale bar: 80 μm.

Supplementary Figure 4 Mixed macrophages in the cochlea with conventional immunofluorescence staining. Images of the representative mixed macrophages in paraffin-embedded sections of the mouse cochlea with cisplatin injection on the day 8. Cochlear mid-modiolar sections were stained by CD68 (M1 marker, green), CD206 (M2-marker, red) and DAPI (blue). Images were taken by a confocal microcopy at 10x and 40× objectives. Boxed regions are magnified in right panels. The upper row shows representative mixed macrophages in the modiolus. The lower row shows M1-like macrophage or CD68^+^ (arrow) and mixed macrophages (arrow heads) in the cochlear bone marrow. Scale bar: 200 μm (low magnification image), 20 μm (high magnified view).

Supplementary Figure 5 Cisplatin caused insignificant damage to the neuronal cells and hair cells. **(A)** β3-tubulin and Iba1 expression in the spiral ganglia and auditory nerves. There was no damage to the neuronal cells of the spiral ganglia or auditory nerves in the different groups. SG, spiral ganglia; AN, auditory nerve. Scale bar: whole cochlea, 500 μm; auditory nerve and spiral ganglia, 90 μm. **(B)** Statistical difference between the neuronal cells in the control and cisplatin (CDDP) groups (*n* = 5). There was no significant difference between the two groups in the spiral ganglia (*P* > 0.9999) and auditory nerve (*P* = 0.0546); ns: not significant by two-way ANOVA with Bonferroni *post hoc* test. The error bars represent standard deviation from the mean. **(C)** Phalloidin staining showing the survival of outer hair cells and inner hair cells of the inner ear in different groups. Images were taken at the basal turn region corresponding to 24–32 kHz. No damage to the inner hair cells was observed in any of the groups. Moreover, the cisplatin group exhibited damage to a few outer hair cells of the basal turn. Red asterisks indicate the damaged cells. Scale bar: 20 μm. **(D)** Statistical analysis of the ratio of the remaining outer hair cells. There was no significant difference in the basal turn between the cisplatin and control groups (*n* = 3) (*P* = 0.6686). Similarly, in the apical and middle turns, there was no significant difference between all groups by two-way ANOVA with the Bonferroni *post hoc* test. The error bars represent standard deviation from the mean. **(E)** Statistical analysis for wave-1 latency at 80dB in control and CDDP groups on day15. Each black dot (control, *n* = 10) and white square (CDDP, *n* = 7) represented wave 1 latency at 8, 24, and 32 kHz. Wave-1 latency at 24 and 32 kHz were significantly increased in CDDP group (*P* = 0.0002 and *P* = 0.0003, respectively), but was not significant at 8 kHz (*P* = 0.0626) by two-way ANOVA with Bonferroni *post hoc* test. The error bars represent standard deviation from the mean.

## Abbreviations

NLRP3, NOD-: LRR- and pyrin domain-containing protein 3
RT-qPCR: reverse transcription-quantitative polymerase chain reaction
mIHC: multiplex immunohistochemistry
ABR: auditory brainstem response
AEC: 3-amino-9-ethyl-carbazole
PBS: phosphate-buffered saline
PFA: paraformaldehyde
FFPE: formalin-fixed paraffin-embedded.
