# Supplementary figures and images for: Multiplex immunohistochemistry reveals cochlear macrophage heterogeneity and local auditory nerve inflammation in cisplatin-induced hearing loss
Source: Front Neurol. 2022 Oct 20;13:1015014. doi: 10.3389/fneur.2022.1015014 (PMC9633043; doi:10.3389/fneur.2022.1015014)

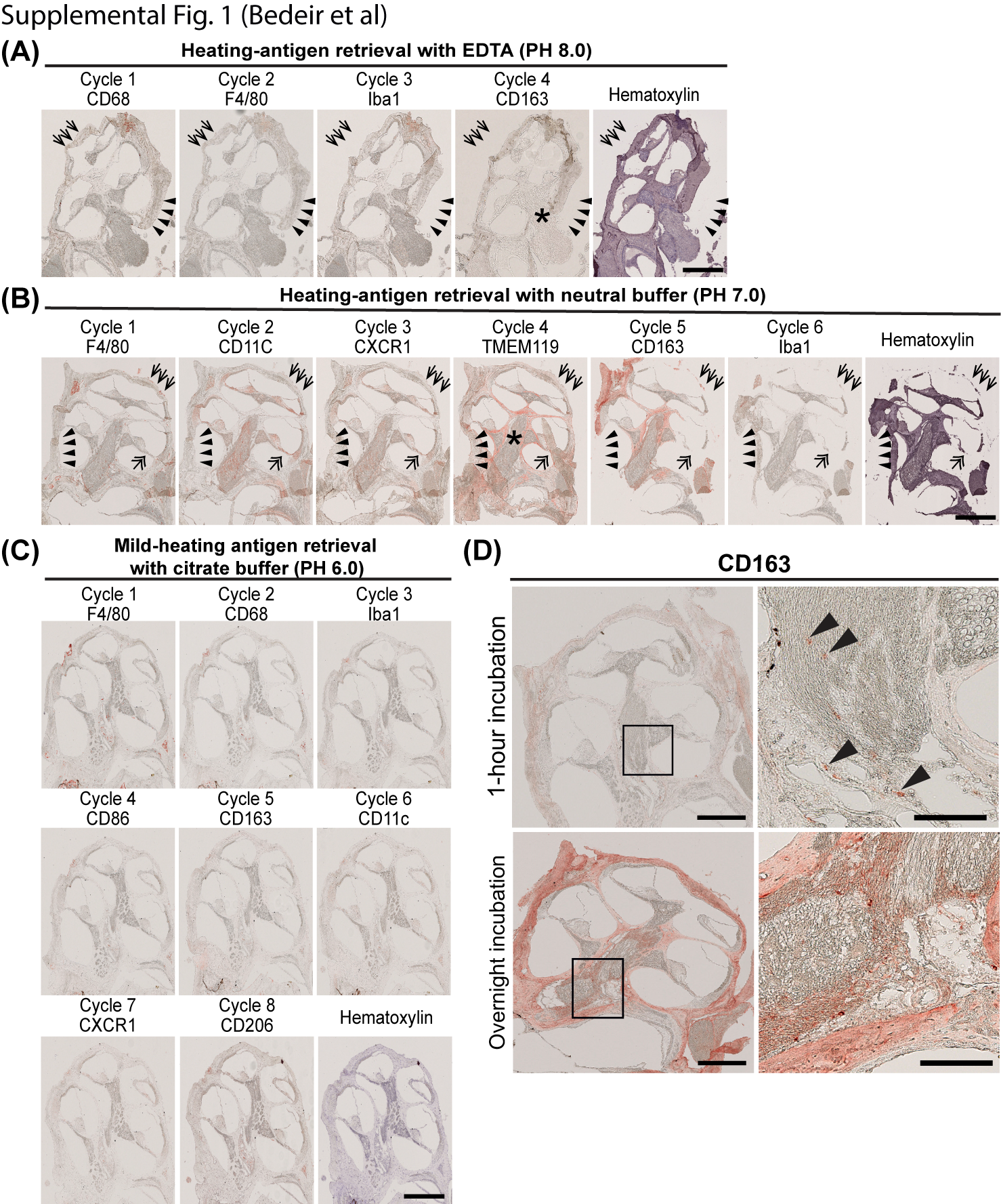

Supplement: Supplementary Figure 1 — Optimization of the iterative antigen-retrieval and primary-antibody incubation steps for the mIHC protocol. Comparison of cochlear tissue damages and background staining among conventional heat-mediated antigen retrieval (AR) at 95°C for 15 min with EDTA (pH 8.0) (A), with neutral buffer (pH 7.0) (B), and mild-heating AR with citrate buffer (pH 6.0) at 70°C for 5 min (C). (A) Minor-tissue damage was detected on the first cycle (arrow) and the section started to be detached after the third cycle (arrow head). Note that intense tissue damage was observed in the modiolus (asterisk), resulted in overstaining of hematoxylin. (B) Tissue-damage was observed from the third cycle (black arrow heads) and most of the cochlea was destroyed after the fifth cycle (arrow and double arrow). Non-specific signaling in the modiolus at the fourth cycle (asterisk) and overstaining of hematoxylin were observed. (C) The cochlear tissue was well-preserved and no major damage was detected up to the end of all the desired staining cycles with clear background. Nuclei were clearly stained by hematoxylin at ninth cycle. (D) Short primary antibody incubation reduced the undesired background. 1-hour incubation at 23°C (upper row) and overnight incubation at 4°C (lower row) with CD163 antibody were shown, and each boxed region was magnified in the right panel. Arrow heads indicated positive signal of CD163 with clear background. Scale bar; (A–C) 500 μm, (D) the whole cochlea 500 μm, the magnified part 200 μm. [file Image_1.tif]

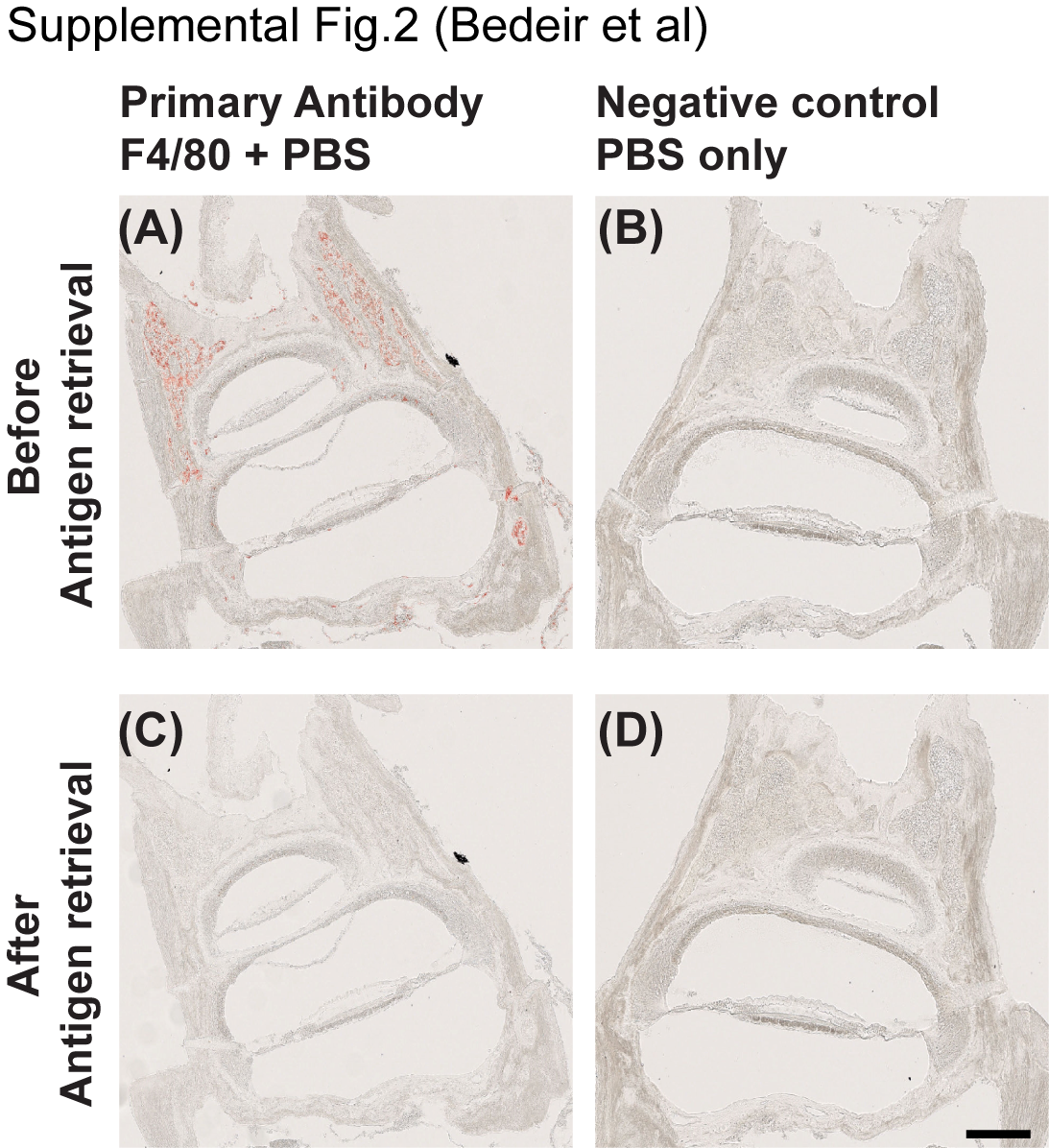

Supplement: Supplementary Figure 2 — Mild antigen retrieval protocol removes antibodies entirely and effectively. (A) Image of the tissue incubated with the F4/80 antibody; the red dots represent a positive signal. (B) Negative control slide incubated with 0.1 M phosphate-buffered saline (PBS); no signal is observed. (C) The same tissue in image (A) after antigen retrieval, followed by incubation with secondary antibody to check for a residual primary antibody signal. The image shows complete stripping of the primary antibody, indicated by the absence of a positive signal. (D) The negative control slide shows no difference after antigen retrieval. Scale bar: 500 μm. [file Image_2.TIF]

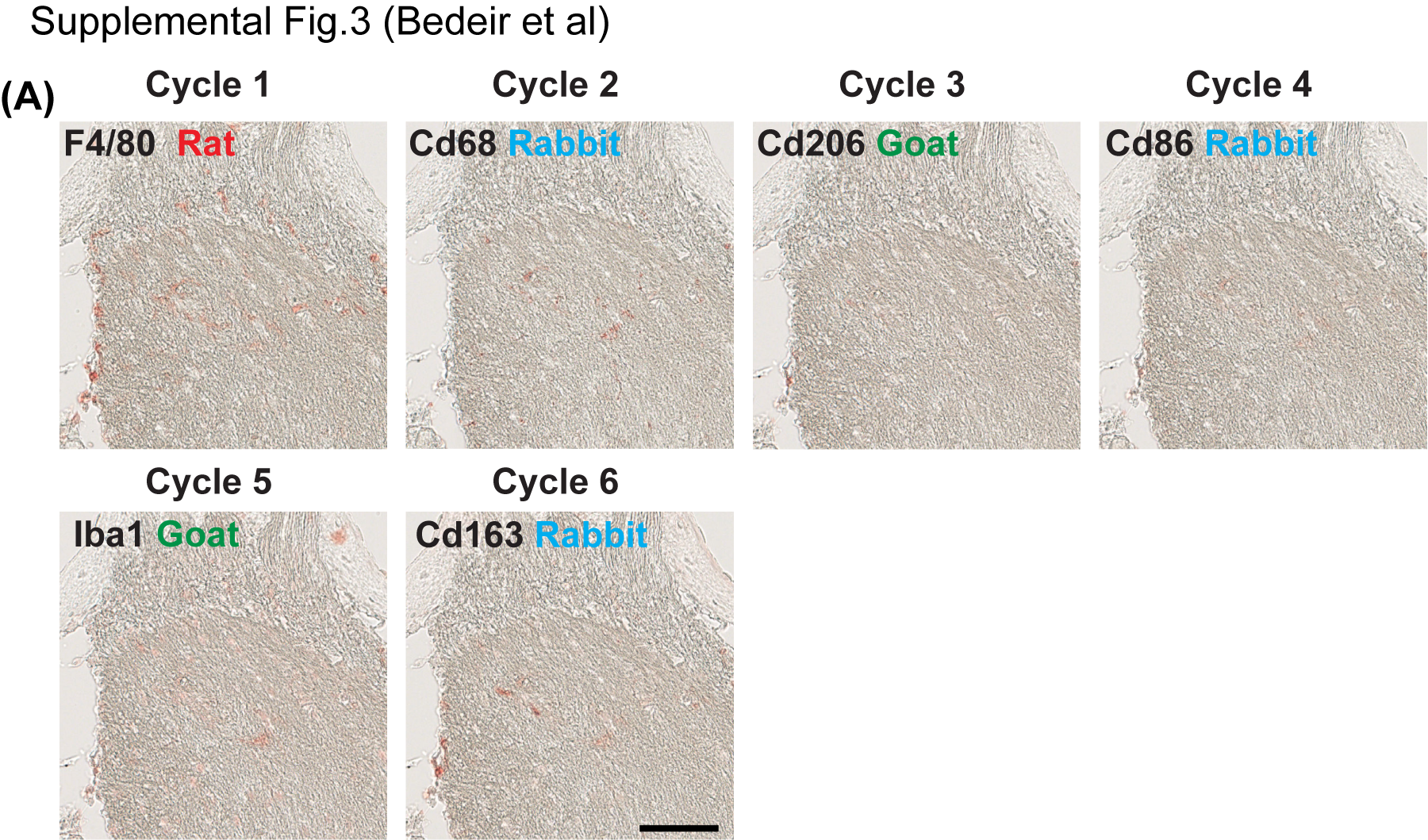

Supplement: Supplementary Figure 3 — Antibodies raised in different host species are used in the sequential immunostaining cycles to confirm the absence of false-positive signals. The images show six immunostaining cycles in the same tissue section. Each cycle of primary antibody incubation was followed by a cycle of incubation with another primary antibody of different species, to avoid a false-positive signal from frequent staining. Scale bar: 80 μm. [file Image_3.TIF]

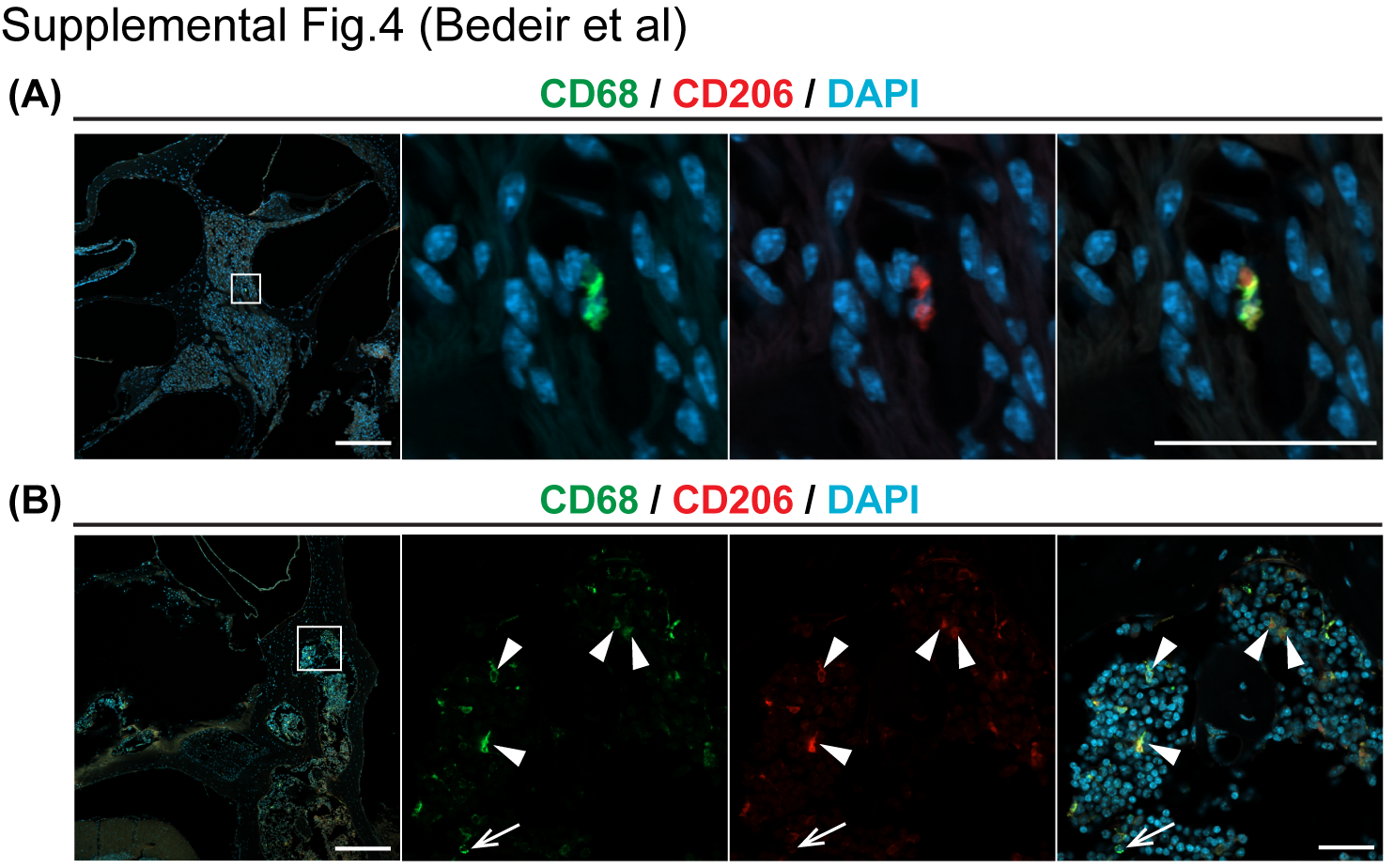

Supplement: Supplementary Figure 4 — Mixed macrophages in the cochlea with conventional immunofluorescence staining. Images of the representative mixed macrophages in paraffin-embedded sections of the mouse cochlea with cisplatin injection on the day 8. Cochlear mid-modiolar sections were stained by CD68 (M1 marker, green), CD206 (M2-marker, red) and DAPI (blue). Images were taken by a confocal microcopy at 10x and 40× objectives. Boxed regions are magnified in right panels. The upper row shows representative mixed macrophages in the modiolus. The lower row shows M1-like macrophage or CD68+ (arrow) and mixed macrophages (arrow heads) in the cochlear bone marrow. Scale bar: 200 μm (low magnification image), 20 μm (high magnified view). [file Image_4.TIF]

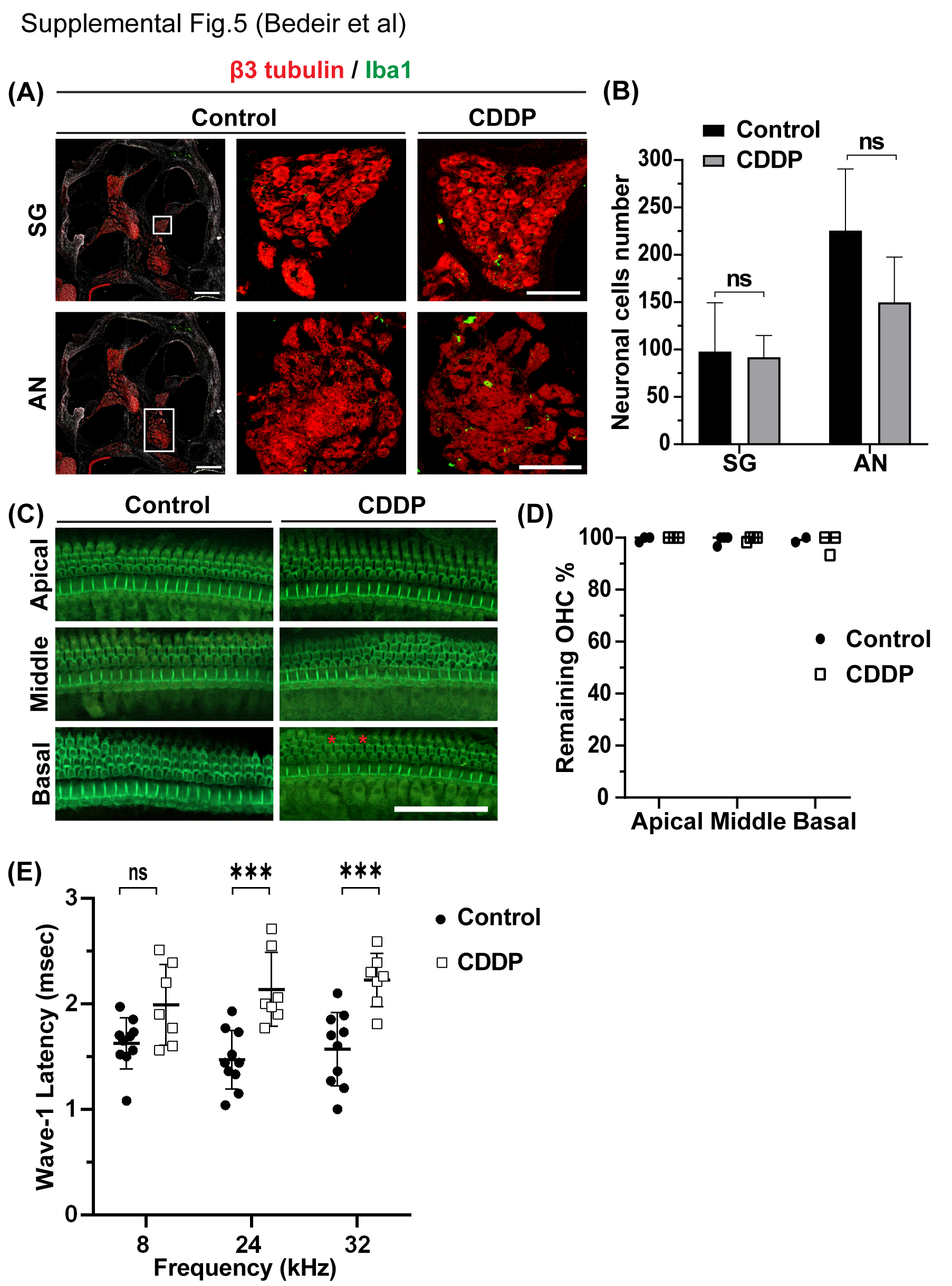

Supplement: Supplementary Figure 5 — Cisplatin caused insignificant damage to the neuronal cells and hair cells. (A) β3-tubulin and Iba1 expression in the spiral ganglia and auditory nerves. There was no damage to the neuronal cells of the spiral ganglia or auditory nerves in the different groups. SG, spiral ganglia; AN, auditory nerve. Scale bar: whole cochlea, 500 μm; auditory nerve and spiral ganglia, 90 μm. (B) Statistical difference between the neuronal cells in the control and cisplatin (CDDP) groups (n = 5). There was no significant difference between the two groups in the spiral ganglia (P > 0.9999) and auditory nerve (P = 0.0546); ns: not significant by two-way ANOVA with Bonferroni post hoc test. The error bars represent standard deviation from the mean. (C) Phalloidin staining showing the survival of outer hair cells and inner hair cells of the inner ear in different groups. Images were taken at the basal turn region corresponding to 24–32 kHz. No damage to the inner hair cells was observed in any of the groups. Moreover, the cisplatin group exhibited damage to a few outer hair cells of the basal turn. Red asterisks indicate the damaged cells. Scale bar: 20 μm. (D) Statistical analysis of the ratio of the remaining outer hair cells. There was no significant difference in the basal turn between the cisplatin and control groups (n = 3) (P = 0.6686). Similarly, in the apical and middle turns, there was no significant difference between all groups by two-way ANOVA with the Bonferroni post hoc test. The error bars represent standard deviation from the mean. (E) Statistical analysis for wave-1 latency at 80dB in control and CDDP groups on day15. Each black dot (control, n = 10) and white square (CDDP, n = 7) represented wave 1 latency at 8, 24, and 32 kHz. Wave-1 latency at 24 and 32 kHz were significantly increased in CDDP group (P = 0.0002 and P = 0.0003, respectively), but was not significant at 8 kHz (P = 0.0626) by two-way ANOVA with Bonferroni post hoc test. The error bars represent standard [file Image_5.TIF]
